# Virtual care pathways for people living with HIV: A mixed‐methods systematic review

**DOI:** 10.1111/hiv.13701

**Published:** 2024-09-17

**Authors:** Hamzah Z. Farooq, Louise Whitton, Chikondi Mwendera, Pip Divall, Sophie J. I. M. Spitters, Jane Anderson, John P Thornhill

**Affiliations:** ^1^ SHARE Collaborative Queen Mary University of London London UK; ^2^ Blizard Institute Queen Mary University of London London UK; ^3^ Department of Infection and Immunity Barts Health NHS Trust London UK; ^4^ Department of Infectious Diseases and Tropical Medicine, North Manchester General Hospital Manchester University Foundation Trust Manchester UK; ^5^ Department of Virology UK Health Security Agency Manchester Manchester UK; ^6^ University Hospitals of Leicester Library University Hospitals of Leicester NHS Trust Leicester UK; ^7^ Wolfson Institute of Population Health Queen Mary University of London London UK

**Keywords:** antiretrovirals, gender equity, global health, HIV, inclusion, telemedicine, virtual care

## Abstract

**Background:**

The COVID‐19 pandemic prompted an unprecedented surge in virtual services, necessitating a rapid shift to digital healthcare approaches. This review focuses on evaluating the evidence of virtual care (VC) in delivering HIV care, considering the complex nature of HIV and the need for tailored‐approaches, especially for marginalized populations.

**Methods:**

A mixed‐methods systematic review was performed with searches on five databases, covering studies from January 1946 to May 2022. Inclusion criteria involved two‐way virtual consultations between healthcare workers and people living with HIV (PLHIV), with detailed descriptions and outcomes. Qualitative and quantitative studies were included, and the risk of bias was assessed using the Newcastle–Ottawa score and Stenfors' framework.

**Results:**

Among 4143 identified records, 26 studies met the criteria, with various models of care described. The majority of studies were observational, and videoconferencing was the primary mode of virtual consultation employed. Quantitative analysis revealed PLHIV generally accept VC, with high attendance rates (87%). Mean acceptability and satisfaction rates were 80% and 85%, respectively, while 87% achieved HIV viral suppression. The setting and models of VC implementation varied, with some introduced in response to COVID‐19 while others were as part of trials.

**Conclusions:**

VC for PLHIV is deemed an acceptable and effective approach and is associated with good virological outcomes. Data on other health outcomes is lacking. The review underscores the importance of diverse models of care, patient choice and comprehensive training initiatives for both staff and patients. Establishing a ‘gold standard’ for VC models is crucial for ensuring appropriate and effective reviews of PLHIV in virtual settings.

## INTRODUCTION

COVID‐19 drove an unprecedented surge in virtual services in all aspects of our daily lives, including health. While remote healthcare existed before the pandemic, the arrival of SARS‐COV‐2 accelerated the pace of change to digital approaches, motivated by the need for social distancing and the need to avoid in‐person contact. The percentage of telehealth claims out of total health claims in the USA was around 25 times higher in January 2022 compared with the pre‐COVID‐19 situation in October 2019. Virtual healthcare suddenly became mainstream in most parts of the world. This en masse shift to virtual care has led to interventions from which a mixed picture of quality and satisfaction is emerging [1]. This review evaluates the evidence for virtual care (VC) in the delivery of HIV care.

The characteristics of HIV – a complex and often stigmatizing condition that disproportionately affects already marginalized and excluded populations – require carefully tailored approaches if remote care is to be acceptable and effective, both for providers and service users. Confidence in maintaining confidentiality, equity of access and individual choice is of particular importance, as is the access to the hardware and data for VC, which may be limited [2]. Poor mental health, language barriers, other comorbidities and polypharmacy are all more common among people with HIV, adding complexity to the consultation [3, 4, 5]. HIV‐specific factors such as assessments for opportunistic infections, HIV‐associated co‐morbidities, monitoring of viral load, and dispensing of medication must also be considered. In addition, delivering a thorough, holistic care approach that considers the individual's overall well‐being should form part of the VC [5].

For most people living with HIV (PLHIV) and their clinical care providers, remote care represents a major departure from “business as usual.” Prior to the SARS‐CoV‐2 pandemic, the model of care globally was largely based on face‐to‐face consultations in a brick‐and‐mortar environment, with the input of large multidisciplinary teams and a major emphasis on confidentiality. In such face‐to‐face settings, which are highly valued by PLHIV [6], clinical and virological outcomes are among the best in the world [4].

Evidence suggests that eHealth interventions can be helpful in HIV care, aiding prevention [7], retention in care [8] and treatment adherence [9]. Work is urgently needed to understand how best to translate eHealth and virtual care into effective person‐centred models of care that meet the complex needs of PLHIV that can be delivered and accessed remotely. Furthermore, understanding the appropriateness and appetite for continued virtual consultations beyond the SARS‐CoV‐2 pandemic will allow for the planning and resourcing of services to implement the best possible HIV care in this new era of healthcare delivery.

There is a lack of evidence on the utility of remote consultation for PLHIV. In this systematic review and narrative synthesis, we review the quantitative and qualitative literature on virtual platforms and virtual consultations for the care of PLHIV.

## METHODS

This mixed‐methods systematic review was performed in accordance with the PRISMA guidelines for performing and reporting systematic reviews and meta‐analyses (Preferred Reporting Items for Systematic Reviews and Meta‐Analyses – PRISMA) [10].

Two independent investigators (HZF and LW) searched five databases (MEDLINE, EMBASE, EMCare, CINAHL and CENTRAL) utilizing the search strategy by an expert librarian (Appendix S1) for studies published from database inception (1 January 1946) to 3 May 2022.

### Search strategy and selection criteria

Two reviewers (HZF and LW) independently screened the titles and abstracts of all studies that were identified through database searches utilizing a systematic screening app [11]. The inclusion criteria were:

- Two‐way virtual consultation between a healthcare worker (HCW) and a PLHIV
- Detailed description of the VC service with described measured outcomes
- Detailed description of the VC service with described models of care.

The exclusion criteria of the review were:

- Non‐HIV care
- No virtual aspect
- One‐way communication (i.e., mobile text messaging only from the HCW)
- Commentary with no detailed description or outcomes of the VC service
- Non‐English language study (as translators were not funded through this study)
- Post‐exposure prophylaxis (PEP) and or pre‐exposure prophylaxis (PrEP) study.

Any conflicting decisions were discussed and referred to a third reviewer (CM or JPT) if a consensus could not be reached.

Both qualitative and quantitative studies were included, with a researcher versed in qualitative methods (CM) screening the qualitative abstracts in conjunction with HZF. Narrative synthesis was used for all qualitative studies.

### Data analysis

We performed quantitative and qualitative data analysis via the Joanna Briggs Institute (JBI) convergent segregated approach to provide integrated findings [12]. For both qualitative and quantitative studies, data were extracted and categorized based on the type of study, mode of consultation, location of the study, and role of the healthcare professional leading the VC for each study.

For quantitative studies, variables of interest extracted were number of participants, type of participants, attendance rate of VC visits (i.e., if the PLHIV attended the VC appointment), did‐not‐attend (DNA) VC rates, acceptability of VC, satisfaction rates of VC, proportion of patients with viral loads less than 50 copies/mL and antiretroviral therapy (ART) adherence. We extracted the data on attendance rate and missed appointments for each quantitative study and utilizing these the completion rate was ascertained based on the attendance of the PLHIV at the virtual care (VC) appointments. Missed appointments for PLHIV who did not attend their VC appointment were also captured for analysis.

For the acceptability of VC, the studies asked participants whether they would like VC if it was available, and they answered either “yes” or “no.”

Data extraction followed a standardized process from each study, utilizing a data capture spreadsheet when quantitative data were available. Descriptive quantitative analysis was performed on the extracted data. For each quantitative study, we quantified the mean and range of the reported measurements in the patient outcomes proxied by attendance, satisfaction, acceptability and DNA rates to show the outcomes for VC consultations.

Systematic analyses were also performed to describe the demographics and location of the study. Descriptive analysis was performed using Jamovi (version 2.2.5) with graphs and figures produced utilizing Tableau Desktop (version 2022.3.17).

### Qualitative narrative synthesis

For each qualitative study, data were collected on the objective, participants, settings (primary health, community, hospital, sexual health clinic, HIV clinic), context of the study, the number of participants, the qualitative tool and methodology utilized, the results and the overall conclusion. Utilizing these, CM performed a narrative synthesis [13] by conducting a thematic analysis as described by Braun and Clarke [14] to summarize and interpret findings from the qualitative studies according to the study aims.

### Models of care

Descriptive narratives were used to describe the implementation models of care of HIV virtual platforms, providing a comprehensive overview of all the literature on VC for HIV care.

### Risk of bias review

All quantitative studies and implementation models of care reviews were assessed for bias using the Newcastle–Ottawa scale (NOS). The NOS evaluates three quality parameters (selection, comparability and outcome) divided across eight specific domains to provide a score for the risk of bias in observational studies [15].

Quality assessment of the qualitative studies was conducted using Stenfors' framework [16] for assessing quality in qualitative research. The framework constitutes five criteria for appraising the trustworthiness of qualitative research including: credibility by exploring how the research findings are conceivable and reliable; dependability which explores the extent to which the research could be replicated in similar conditions; confirmability that shows a clear link or relationship between the data and the findings; transferability assesses the extent to which the findings may be transferred to another setting, context or group; and reflexivity is the continual process of engaging with and articulating the place of the researcher and the context of the research.

## RESULTS

**FIGURE 1:**
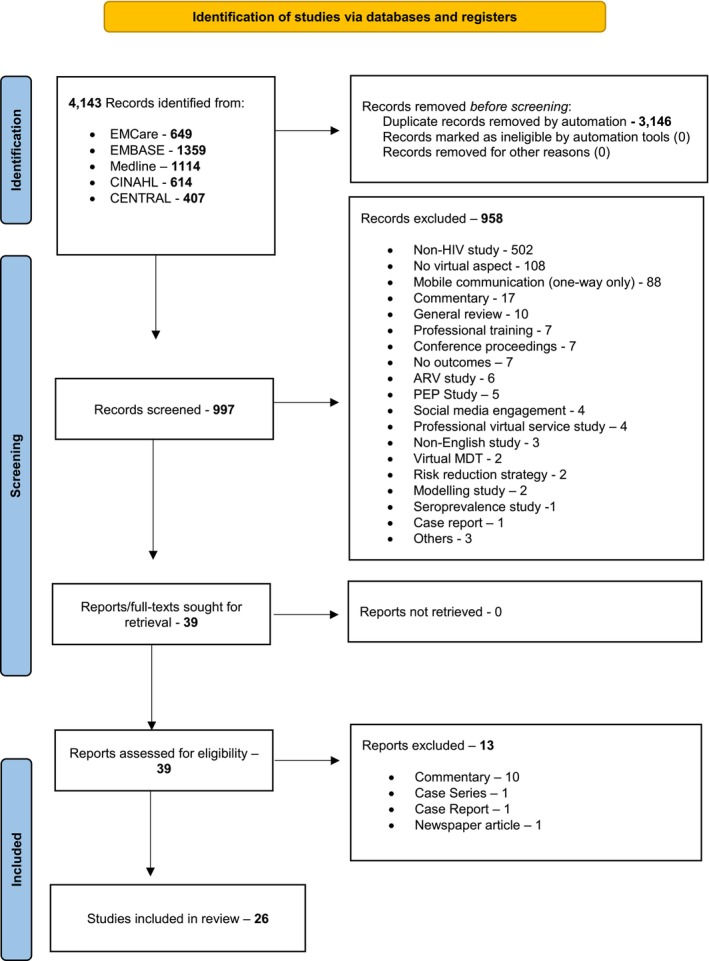
PRISMA (Preferred Reporting Items for Systematic Reviews and Meta‐Analysis) diagram of systematic review screening process [11]. Further information: http://www.prisma‐statement.org/. ARV, antiretroviral; MDT, multidisciplinary team; PEP, post‐exposure prophylaxis.

A total of 4143 records were identified from the aforementioned five databases with 3146 records removed as duplicates. Some 997 records were screened with 39 full texts retrieved and assessed for eligibility. Of these, 26 studies [17, 18, 19, 20, 21, 22, 23, 24, 25, 26, 27, 28, 29, 30, 31, 32, 33, 34, 35, 36, 37, 38, 39, 40, 41, 42] were included, with 13 excluded due to the defined reasons (Figure 1).

### Study characteristics

Of the 26 studies included, 15 were quantitative studies, four qualitative studies, one mixed qualitative and quantitative study, with the remaining six descriptive implementation science studies or VC “Models of HIV care” (Tables 1, 2, 3, 4, 5).

**TABLE 1:** Included studies and study types.

Study	Type of study	Reference
Auchus et al. (2021)	Quantitative and Model of Care	[17]
Badowski and Nyberg (2012)	Quantitative and Model of Care	[18]
Brody et al. (2021)	Model of Care	[19]
Coppock et al. (2021)	Model of Care	[20]
Dandachi et al. (2020)	Quantitative	[21]
Dandachi et al. (2020)	Model of Care	[22]
Diedrich et al. (2021)	Systematic Review/Quantitative	[23]
Drummond et al. (2017)	Model of Care	[24]
El‐Nahal et al. (2022)	Quantitative and Model of Care	[25]
Guaraldi et al. (2021)	Quantitative	[26]
Hoberg et al. (2018)	Quantitative and Model of Care	[27]
Jain et al. (2019)	Quantitative and Model of Care	[28]
John et al. (2016)	Quantitative and Model of Care	[29]
Junkins et al. (2021)	Quantitative, Model of Care and Qualitative	[30]
León et al. (2011)	Quantitative and Model of Care	[31]
Marent and Henwood (2021)	Model of Care and Qualitative	[32]
Palfai et al. (2020)	Quantitative and Model of Care	[33]
Reynolds et al. (2016)	Model of Care	[34]
Rogers et al. (2020)	Model of Care	[35]
Saberi et al. (2013)	Model of Care and Qualitative	[36]
Saifu et al. (2012)	Quantitative and Model of Care	[37]
Salgado et al. (2021)	Quantitative and Model of Care	[38]
Trepka et al. (2022)	Quantitative and Model of Care	[39]
Turner et al. (2019)	Qualitative	[40]
Wood et al. (2020)	Quantitative and Model of Care	[41]
Yelverton et al. (2021)	Qualitative	[42]

**TABLE 2:** Findings of studies – quantitative studies and models of care.

Summary of Findings – Models of Care													
Study Name	Location	Intervention initiated as pilot/intervention study/RCT?	Political/systemic context	Types of telehealth used	Patient in controlled setting (e.g., local clinic)	Care provided by telehealth to patient	Used as alternative to some usual care?	Face‐to‐face visit standard part of protocol?	Telehealth exclusion criteria described	Telehealth implemented on an opt‐in basis	Staff training described	Patient training described	Other steps made to counteract digital exclusion
Trepka et al. (2022) [23]	Florida, USA	No	COVID‐19 pandemic	Phone, email, SMS	No	Case manager, Clinician, Mental health, Substance misuse work	√	X	X	X	X	X	X
El‐Nahal et al. (2022) [9]	Baltimore, USA	No	COVID‐19 pandemic	Video or phone	No	All care	√	X	Acute concerns/initiating ART	X	√	√	X
Junkins et al. (2021) [14]	Alabama, USA	Yes	Health inequities in African‐American women with depression	Video	Yes	Mental health	X	X	Significant substance abuse/other mental health disorder, Recent CBT	√	X	X	X
Salgado et al. (2021) [22]	Georgia, USA	No	Very rural area (Expansion in COVID‐19 pandemic)	Digital examination	Yes (No during COVID‐19 pandemic)	Clinician	√	X	X	X	√	X	X
Auchus et al. (2021) [1]	San Francisco, USA	No	COVID‐19 pandemic	Unclear	No	Clinician, Nursing, Social work, Nutrition	√	X	X	X	X	X	X
Marent et al. (2021) [16]	5 centres, Europe	Yes	EmERGE study	Two‐way messaging	No	Clinical, Nursing, Psychology	√	Yearly	Patients not classed as ‘stable’	√	X	X	X
Brody et al. (2021) [3]	Boston, USA	No	COVID‐19 pandemic, Homeless patients	Phone	No	All care with extra case management	√	X	X	X	X	X	√
Coppock et al. (2021) [4]	Pennsylvania, USA	No	COVID‐19 pandemic	Two‐way messaging	No	Clinical, Nursing, Case manager	√	X	X	X	√	X	X
Rogers et al. (2020) [19]	New England, USA	No	COVID‐19 pandemic	‘Web conferencing’	No	Substance use treatment	√	X	X	√	√	√	√
Dandachi et al. (2020) [6]	Missouri, USA	No	COVID 19 pandemic	Phone if no smartphone	No	Basic HIV care. Not described in detail	√	X	X	X	X	X	X
Palfai et al. (2020) [17]	Boston, USA	Yes	Pilot, For chronic pain and addiction treatment	Video	No	Intervention to reduce heavy drinking and chronic pain	X	Initial visit	Non‐English speaking, Not engaged in care, ‐ history of bipolar disorder, schizophrenia, or complicated alcohol withdrawal, ‐ current psychosocial treatment, ‐ anticipated surgery	√	X	√	√
Wood et al. (2020) [25]	Philadelphia, USA	No	COVID‐19 pandemic, Adolescent healthcare including HIV care described	Video	No	Clinician, Nursing, Behavioural health, Dietician, SW, +/–Interpretation	Yes	No	‐ No electronic health record account, ‐No mobile device or computer, ‐No internet access or private area, ‐LARC, ‐Severe gynae issue, ‐ Significant risk of mental health decline, ‐ Eating disorder with concern for medical instability.	X	√	X	X
Jain et al. (2019) [12]	Alabama, USA	No	Rural Alabama, in context of rising HIV incidence in 2011	Video, with in‐person nurse facilitation, Digital examination	Yes	Clinicians, SW, Case management, pharmacy, mental health +/− translation services	√	Initial visit	X	√	√	X	X
Horberg et al. (2018) [11]	Columbia, Maryland and Virginia, USA	No	Increasing use of virtual care at organization described (Not for profit)	Phone, Email	No	Clinician	√	Yearly	X	X	X	X	X
Drummond et al. (2017) [8]	Southern USA	Yes	Effectiveness trial, off‐site collaborative MDT for mental health, Veterans	Phone	No	Depression care manager	X	X	‐No access to telephone, ‐Suicidal ideation, ‐Significant cognitive impairment, ‐History of bipolar disorder/ Schizophrenia	√	X	X	X
John et al. (2016) [13]	South Nigeria	Yes	Trial, to engage in non‐disclosed youth	Phone, SMS, WhatsApp	No	Weekly health promotion call, Hotline	No	No	X	√	X	X	X
Reynolds et al. (2016) [18]	South India	Yes	Control trial, to improve mental health in women new to ART	Phone	No	1–2 weekly health promotion call by nurse, Hotline	No	No	Unusual languages, Exclusion at CI discretion, Suicidal ideation, ‐Unable to be contacted by mobile	√	√	√	√
Saberi et al. (2013) [20]	San Francisco, USA	Yes	Pilot study to engage African‐American youths	Video	Yes	Clinical pharmacist, HIV education	No	No	Excluded severe cognitive impairment and psychosis	√	X	X	X
Badowski et al. (2012) [2]	Illinois, USA	No	Prison population	Video with in‐person nurse facilitation, Digital examination	Yes	Clinician pharmacist, Case manager	Yes	No	X	X	X	X	X
Saifu et al. (2012) [21]	Los Angeles, USA	No	Rural areas, Veterans	Video with in‐person nurse facilitation	Yes	Clinician	Yes	No	Unstable patients (referred into clinic by usual HIV clinician)	X	X	X	X
León et al. (2011) [15]	Barcelona, Spain	Yes	Trial to improve follow‐up for stable patients	Video, Two‐way messaging	No	Clinician, Nursing, Psychology, SW, Pharmacy	Yes	2 out of 4 visits still in person	Do not own a phone, HIV not stable or VL > 20 000, CD4 < 200, pregnancy, Clinician discretion	√	X	X	X

Abbreviations: ART, antiretroviral therapy; CBT, cognitive behavioural therapy; MDT, multidisciplinary team; RCT, randomized controlled trial; SW, social‐worker, define; VL, viral load.

**TABLE 3:** Summary of findings – quantitative studies and measurable outcomes.

Summary of Findings – Quantitative studies and measurable outcomes										
Study Name	Type of Study	Intervention Name	Mode of Consultation	Back up Consultation method	Location	Name of consultation tool	Number of participants	Type of participants	Completed appointments, *n* (%)	Cancelled/DNA, *n* (%)
Auchus et al. (2021) [1]	Cohort	no data	Video‐Conferencing	Telephone	USA	Zoom	1418	General PLHIV	846 (60.9%)	554 (39.1%)
Badowski et al. (2012) [2]	Cohort	no data	Video‐Conferencing	Telephone	USA	Unknown	700	Incarcerated PLHIV	700 (100%)	0 (0%)
Dandachi et al. (2020) [5]	Cohort	no data	Video‐Conferencing	Telephone	USA	Unknown	371	General PLHIV	No data	No data
León et al. (2011) [15]	RCT	Virtual Hospital	Video‐Conferencing	Telephone	Spain	Virtual Hospital	83	General PLHIV	76 (92%)	7 (8%)
El‐Nahal et al. (2022) [9]	Cohort	no data	Video‐Conferencing	Telephone	USA	Unknown	1780	General PLHIV	1600 (90%)	180 (10%)
Guaraldi et al. (2021) [10]	Commentary	no data	Video‐Conferencing	Telephone	Global	No data	No data	General PLHIV	No data	No data
Hoberg et al. (2018) [11]	Cohort	no data	IP and Telephone	No data	USA	No data	3114	General PLHIV	No data	No data
Hoberg et al. (2018) [11]	Cohort	no data	IP and Email	No data	USA	No data	3114	General PLHIV	No data	No data
Hoberg et al. (2018) [11]	Cohort	no data	IP and Telephone and Email	No data	USA	No data	3114	General PLHIV	No data	No data
Jain et al. (2019) [12]	Descriptive Implementation Review/Model of care	Alabama eHealth	Video‐Conferencing	Telephone	USA	Alabama eHealth	240	General PLHIV	183 (76.30%)	57 (24%)
John et al. (2016) [13]	Cohort	no data	Text‐Messaging	No data	Nigeria	No data	19	General PLHIV	14 (74.60%)	5 (25.40%)
Junkins et al. (2021) [14]	RCT	no data	Video‐Conferencing	No data	USA	No data	22	Women PLHIV	21 (95%)	1 (5%)
Palfai et al. (2020) [17]	Pilot study	no data	Video‐Conferencing	No data	USA	No data	8	General PLHIV	7 (88%)	1 (12%)
Saifu et al. (2012) [21]	Cohort	VAGLAHS	Video‐Conferencing	Telephone	USA	No data	43	PLHIV and PLHCV	30 (70%)	13 (30%)
Salgado et al. (2021) [22]	Cross‐sectional	Ryan White Programme	Video‐Conferencing	Telephone	USA	Cisco WebX Services	1041	General PLHIV	1041 (100%)	0 (0%)
Trepka et al. (2022) [23]	Cross‐sectional	Ryan White Programme	Video‐Conferencing	Telephone	USA	Cisco WebX Services	114	General PLHIV	114 (100%)^b^	0 (0%)
Wood et al. (2020) [25]	Cross‐sectional observational study	no data	Video‐Conferencing	Telephone	USA	Epic Systems	331	Adolescent PLHIV	282 (85%)	49 (15%)

Summary of Findings – Quantitative studies and measurable outcomes										
Study Name	Frequency	Acceptability to use TeleHIV, *n* (%)	Satisfaction rate, *n* (%)	Nurse/doctor led	ART delivery	Access to bloods	Health Promotion	STI Screening	VL < 50	ART Adherence
Auchus et al. (2021) [1]	3‐Monthly	N/A	108 (53.1%)^a^	No data	Unknown	Unknown	Unknown	No data	Unknown	Unknown
Badowski et al. (2012) [2]	6‐weekly	N/A	N/A	Both	For 1 month supply	Bloods on appointment day	HIV Adherence counselling	No data	Unknown	Unknown
Dandachi et al. (2020) [5]	No data	57%	N/A	No data	no data	no data	no data	No data	67%	67%%
León et al. (2011) [15]	6‐Monthly	65 (82%)	65 (82%)	Both	6‐Monthly and posted	Blood test 15 days pre‐appointment	HIV Adherence counselling, Psychiatry services	Yes	Unknown	95%
El‐Nahal et al. (2022) [9]	6‐Monthly	N/A	N/A	Both	Unknown	Unknown	Unknown	Yes	92%	Unknown
Guaraldi et al. (2021) [10]	12‐Monthly	N/A	N/A	Both	6‐Monthly and posted	Yearly	HIV Adherence counselling, Psychiatry services, frailty, cognitive impairment	Yes	No data	No data
Hoberg et al. (2018) [11]	No data	No data	No data	No data	No data	No data	No Data	No data	86%	No Data
Hoberg et al. (2018) [11]	No data	No data	No data	No data	No data	No data	No Data	No data	91.60%	No Data
Hoberg et al. (2018) [11]	No data	No data	No data	No data	No data	No data	No data	No data	90.70%	No data
Jain et al. (2019) [12]	6‐Monthly	No data	No data	No data	No data	No data	No data	No data	75%	96.90%
John et al. (2016) [13]	No data	14 (74.6%)^c^	14 (74.6%)^c^	Doctor	No data	No data	No data	No data	No data	No data
Junkins et al. (2021) [14]	3‐Monthly	19 (85.1%)^c^	19 (85.1%)^c^	Psychologist	No data	No data	No data	No data	85.70%	76.2%%
Palfai et al. (2020) [17]	No data	No data	No data	Psychologist	No data	No data	No data	No data	No data	No data
Saifu et al. (2012) [21]	No data	41 (97%)^c^	41 (97%)^c^	Doctor	No data	No data	No data	No data	No data	No data
Salgado et al. (2021) [22]	No data	No data	No data	Doctor	No data	No data	No data	No data	91.40%	99.40%
Trepka et al. (2022) [23]	No data	No data	No data	Doctor	No data	No data	No data	No Ddata	No data	No data
Wood et al. (2020) [25]	No data	No data	No data	Doctor	No data	No data	No data	No data	No data	No data

Abbreviations: IP, In‐person; N/A, not available; PLHCV, person living with hepatitis C; PLHIV, person living with HIV; VC, virtual care.

^a^ Only 202 patients surveyed out of 1418 participants.

^b^ Primarily investigated HIV care access during the COVID‐19 pandemic.

^c^ Satisfaction with VC used as a surrogate marker for acceptability if acceptability data not available.

**TABLE 4:** Summary of findings – qualitative studies.

Reference	Objective	Participants and setting/context	Methods	Results	Conclusion
Saberi et al. (2013)	This was a pilot study examining the feasibility and acceptability of the use of telehealth in a controlled clinical setting for medication counselling sessions with HIV‐positive African‐American youth.	Included participants who identified as African‐Americans aged between 18 and 29 years that were on antiretroviral medication for at least 30 days. The study was conducted in San Francisco, USA. Specifically, focused on all HIV clinic teams at the University of California, San Francisco (UCSF) HIV clinics, and other San Francisco Bay Area HIV clinics.	The study provided an initial telehealth intervention through an approved desktop videoconferencing software application (Movi). Participants engaged with a healthcare provider, in a controlled clinical setting, on a face‐to‐face videoconferencing counselling session followed by a 30‐min in‐person semi‐structured qualitative interview by a trained researcher.	Included 14 participants, who were 86% male. Participants stated to like telehealth and indicated that it supported privacy, convenience, improved communication, knowledge, motivation and skills to minimize non‐adherence.	The study concluded that the intervention was feasible and acceptable in a clinical setting, but future studies should explore the feasibility of conducting similar sessions with participants in their home settings.
Yelverton et al. (2021)	This study aimed to understand telehealth utilization for HIV care services in South Carolina, identify barriers to telehealth during COVID‐19, and investigate strategies to facilitate remote HIV care delivery.	Focused on health providers (management personnel) from eight HIV‐related facilities including an academic medical centre, local AIDS service organizations, and the South Carolina state public agency. The study was conducted in South Carolina, USA, which is ranked in the US top 10 for the number of annual HIV/AIDS cases.	Semi‐structured in‐depth interviews were conducted with management personnel, who were as assumed to have a broad knowledge of their organizations' daily operations.	11 interviews were conducted, and participants stated that barriers included technology challenges, digital literacy, client/provider experiences low socioeconomic status of client population. Promoting telehealth included client empowerment and provider training to improve organizational readiness.	By focusing the study beyond medical HIV care services, the study contributed to new findings about non‐medical HIV services and their challenges with telehealth since the onset of COVID‐19. It calls on more efforts to promote telehealth and remove the barriers by implementing inclusive multilevel strategies.
Junkins et al. (2021)	This pilot study aimed to test the feasibility and acceptability of a telemedicine‐administered, culturally adapted cognitive behavioural therapy for depression and antiretroviral therapy adherence (CBT‐AD) approach using videoconferencing among African‐American women living with HIV in the rural Deep South in USA.	Participants were African‐American women living with HIV in the Deep South (Alabama, Georgia, Louisiana, Mississippi, North Carolina and South Carolina) in the USA. The African‐American women are highly impacted by HIV with incident rates comparable to those among women in sub‐Saharan Africa and the prevalence of depression among adults is highest among these women when compared with White women.	The CBT‐AD intervention or supportive psychotherapy, both of which were delivered via videoconference, were randomly allocated to the participants. While mixed methods were used to collect various data with individual interviews conducted to gain deeper insight on acceptance of the interventions and use of telemedicine for their counselling needs. Participants completed 10–12 weekly therapy sessions and 6‐month follow up.	11 women were randomly recruited to each intervention. Both arms saw a significant reduction in depression symptoms, and measures of antiretroviral medication adherence showed good levels of adherence. High levels of satisfaction with the intervention's components were seen, and videoconferencing was regarded as equally effective as in‐person counselling.	The study demonstrated that telemedicine‐administered CBT‐AD is feasible and highly acceptable among women living with HIV.
Turner et al. (2019)	The study aimed at exploring if, and how, the Technology Readiness and Acceptance Model (TRAM) could be used to explain the willingness of men to take part in video‐groups.	Included HIV‐positive men aged 18 years and above who reported having sex with other men. The study population was recruited from South‐eastern United States. This population faces many barriers of engagement thus their views were critical in shaping engagement interventions.	Eligible participants were included in a semi‐structured survey that explored views on video‐groups conducted through private venue, or videophones, or on their own devices at home. Participants that expressed willingness in the interventions were further asked to explain their response.	106 participants took part in this study. There was a general willingness to participate in video‐groups among the participants. Issues of insecurity, discomfort, perceived usefulness, ease of use, innovativeness, privacy and readiness were among the factors influencing willingness.	The study found a general willingness to use video‐groups for HIV‐related programmes irrespective of location and that a modified TRAM may be useful when shaping factors most important to men living with HIV participating in telehealth.
Marent and Henwood (2021)	The study aim was to assess the implications of the digital care pathway by understanding the experiences of both patients and health providers on the opportunities and restrictions of different forms of doctor–patient interactions.	The study included stable HIV patients and health professionals who were using a digital health platform for 6 months in the EmERGE study. The study was implemented across five clinical sites of Barcelona, Brighton, Lisbon, Zagreb and Antwerp in the European Union.	The study involved co‐designed workshops and interviews with both patients and health professionals. The focus was to capture experiences during their interactions when using the EmERGE digital health platform.	There were eight co‐designed workshops, 34 patient interviews and 32 interviews with health professionals.	The study built on a framework of patient–‐doctor relationship when using digital platforms and guides practical reflections on when and how different consultation types may be integrated or complement each other within the care pathways.

**TABLE 5:** Qualitative assessment of reviewed studies.

Reference	Credibility	Dependability	Confirmability	Transferability	Reflexivity
Saberi et al. (2013)	The study methodology is clearly outlined. However, they did not present the tentative number of HIV‐positive African‐American who access the clinics to provide an idea of whether the sample was adequate for the study. They did not provide their analytical theory basis. But they presented a fair balance of negative views.	The authors provide a clear methodology that can be replicated in other settings, but they do not explicitly describe how data were managed and analysed.	There is a clear link between their data and findings as they provide detailed description and the use of quotations.	The researchers provide a detailed description of the context in which the research was performed and how it shaped the findings. However, the extent of the target population was not provided to determine if data were sufficient from their study.	There is slight description of the researcher who conducted the interview that they were trained. Other than that, not much is provided to determine how they influenced the process of data collection.
Yelverton et al. (2021)	The authors clearly provide their methodology and efforts to reach out to the target population in all the 27 HIV‐related facilities in South Carolina. Data analysis was guided by a Grol and Wensing (ref) conceptual framework.	The methods provided can be replicated in other settings and clear processes of data management have been outlined.	The findings clearly relate to the methods used and quotations have been used to assert participants' views.	The context in which the study was conducted is clearly described and the reason for selecting the targeted participants. However, no efforts were made to explore views of service users which could have provided further interesting findings. In addition, the sample size seems small that limits generalizability.	There is no description/reflexivity for those conducting the interviews but only mentions about the interviews being conducted by experienced interviewers.
Junkins et al. (2021)	The study provides a clear background and the extent of the problem in the study population. The methodology is explicit and can be replicated. However, the sample size is small. The qualitative part is justifiable to collect in‐depth views that were thematically analysed to highlight the feasibility and acceptability of the interventions under study. Opposing views are also presented in the findings.	The authors provide clear methodological procedures that can be replicated in other settings. They explain how qualitative data were handled and analysed.	The qualitative part of the study was important in getting deeper understanding of the interventions and the findings complement the quantitative part with quotations affirming participants' views.	The context and study participants are clearly described. But the sample size in each group is small which creates a challenge to generalize the findings beyond the sample in the study.	The authors do not describe who conducted the interviews and analysis hence no reflexivity highlighted.
Turner et al. (2019)	The authors have been explicit in narrating the research process and the analysis plan according to the theoretical framework used. Data were thematically analysed, and the findings have been presented according to the framework used in data collection. The sample size is large enough to provide depth in the data collected.	The study methodology and analysis plan have been provided that can guide replication of the study by other researchers.	The results are presented in a structured format based on the framework used during data collection and quotes have been used to affirm the views of the participants.	The study population is clearly described including the challenges they face which led to the purpose of carrying out this study. However, the study area is not clearly presented as there is no mention of where exactly the participants came from.	The authors provide little insight on who was conducting the interviews as they are only presented as experienced researchers.
Marent and Henwood (2021)	The authors provide the theoretical basis of the study while building on existing frameworks, they develop a clear methodology to capture the views of both patients and health providers. The sample sizes of both patients and health professional are adequate to provide rich data. By using two data collection strategies and analysis through the grounded theory, the researchers provide triangulated findings that are reliable.	The methodology is well presented for others to replicate although the process of co‐designing the workshop is not provided for an insight on how they were conducted.	The findings are presented based on the framework used during data collection while analytical approach justifies data presentation with quotations supporting participants' views.	The context in which this study was conducted is clearly presented and the EmERGE study, in which this evaluation is embedded, is also described. This provides detail about the context in which the study was conducted. The sample sizes of each study group are adequate to support generalizability of the findings.	The authors do not provide insight of those conducting this study to understand their perspective in influencing how the study was conducted.

From a data analysis perspective for all 26 studies, there were 20 studies describing HIV‐VC models of care, 16 described the VC pathway with extractable measurable quantitative outcome data (Tables 1 and 6) and five were qualitative studies (Tables 4 and 5). Of the 16 studies (16/26) with extractable measurable quantitative outcome data (Tables 1 and 6), one was excluded as it was a systematic review from which no additional studies were found [23], resulting in a total of 15 studies with quantitative data. Twenty studies described HIV models of care in which virtual HIV pathways had been implemented, of which one was excluded as it was a published trial protocol of a study which did not measure HIV‐VC outcomes [34].

**TABLE 6:** Summary of data analysis.

Parameter	Total studies	Total number of participants (%)
Completed visits	12/15	4914/5799 (84.74%)
DNA/cancelled	12/15	687/5619 (11.85%)

		Mean
Acceptability	5/15	79.54% (IQR 16.45%, SD 9.98%)
Satisfaction rate	5/15	85% (IQR 10.5%, SD 14.5%)
VL < 50 copies/mL	8/15	87% (IQR 8.43%, SD 91.19%)
Adherence	5/15	86.90% (IQR 21.95%, SD 20.60%)

		Range
Frequency	7/15	6‐weekly to 12‐monthly
ART delivery	3/15	1‐monthly to 6‐monthly/posted

Abbreviations: ART, antiretroviral therapy; DNA, did not attend; IQR, interquartile range; SD, standard deviation; VL, viral load.

### Quantitative studies results

#### Quantitative study characteristics

**FIGURE 2:**
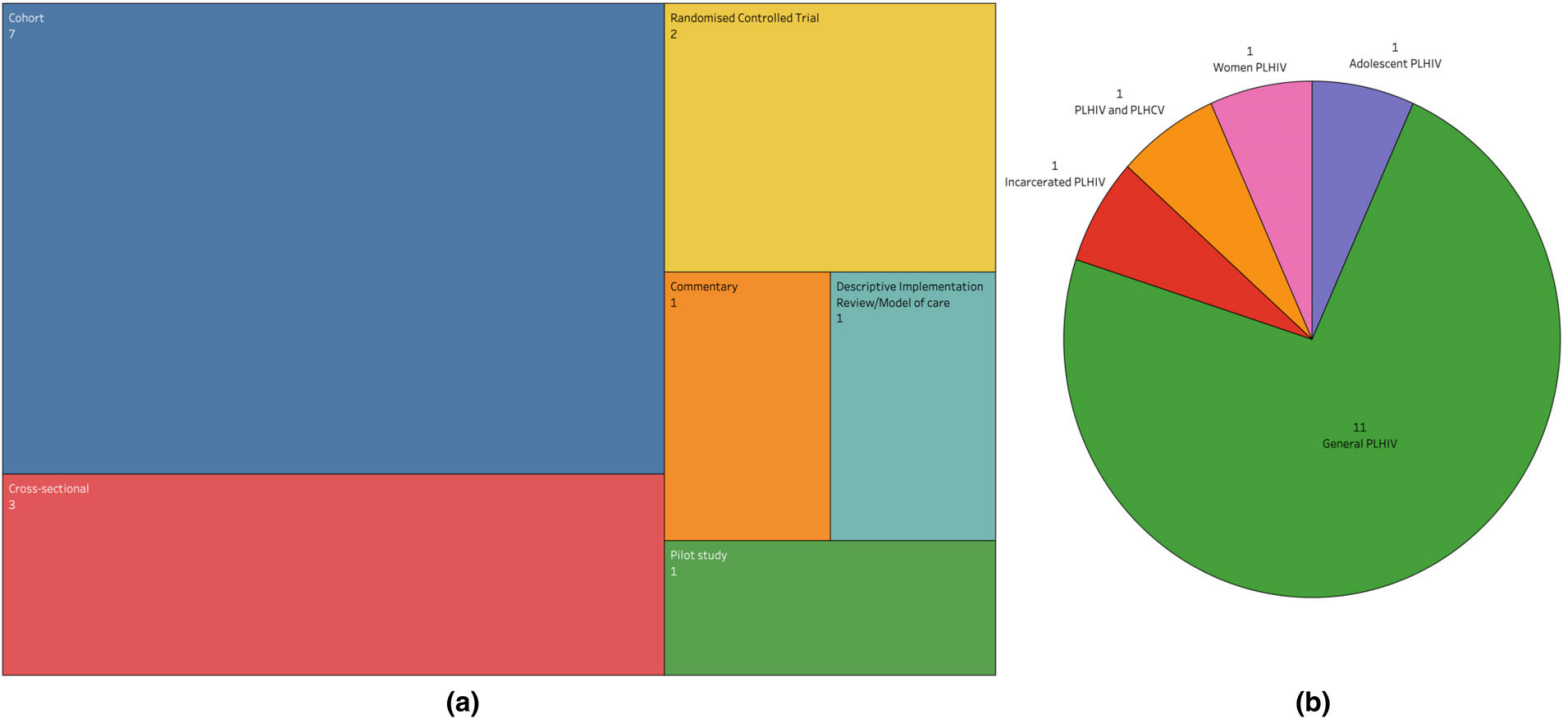
(a) Types and number of studies included in the systematic review and (b) types of participants of primary focus of included HIV virtual care studies. PLHCV, person/people living with hepatitis C; PLHIV, person/people living with HIV.

The majority of studies were observational studies (9/15) with seven cohort studies, three cross‐sectional studies with one pilot study, implementation review and a descriptive commentary. There were only two randomized controlled trials (RCTs) [30] (Figure 2), including one mixed‐methods RCT, which aimed to demonstrate the feasibility and acceptability of implementing cognitive behavioural therapy (CBT) for depression treatment via videoconferencing among African‐American women living with HIV in the USA. The second RCT was a prospective study performed over 2 years, comparing standard care (clinic care) with the HIV‐VC platform Virtual Hospital Care in Spain. PLHIV were randomized to be monitored through a virtual hospital (Arm I) or standard care at the day hospital (Arm II). After 1 year of follow‐up, patients switched their care to the other arm.

**FIGURE 3:**
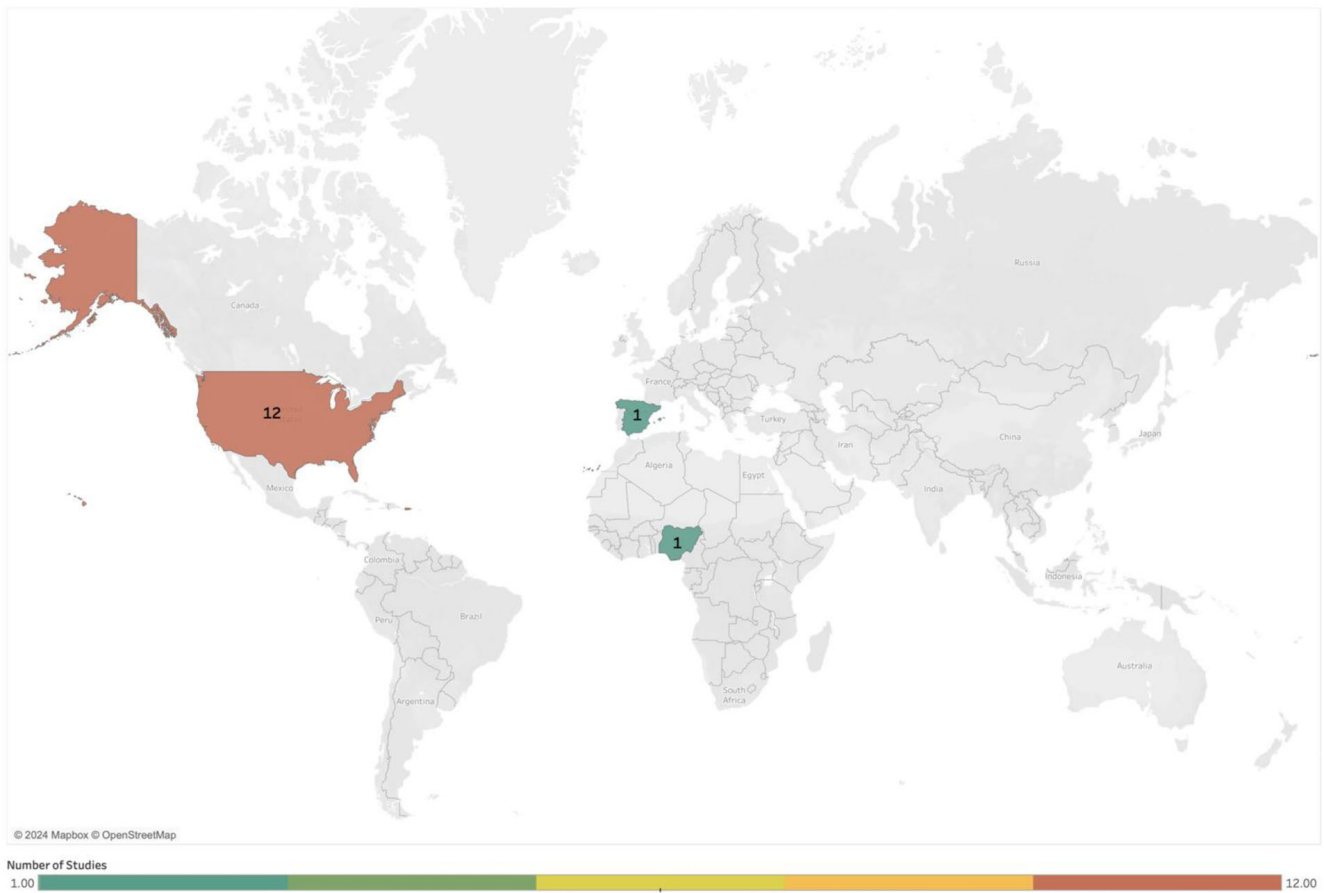
Map of location of all included studies. Greyed‐out sections indicate that no included studies were conducted in these countries.

The clinical setting of studies included the delivery of general HIV virtual care (*n* = 11) with one describing VC for adolescent PLHIV (*n* = 1), incarcerated PLHIV (*n* = 1), women‐only PLHIV (*n* = 1) and PLHIV with chronic hepatitis C infection (*n* = 1). Only one study was based primarily in the Global South, with 93.3% (14/15) based in the Global North and primarily in the USA (14 studies; Figure 3).

#### Characteristics of modes of HIV‐VC

**FIGURE 4:**
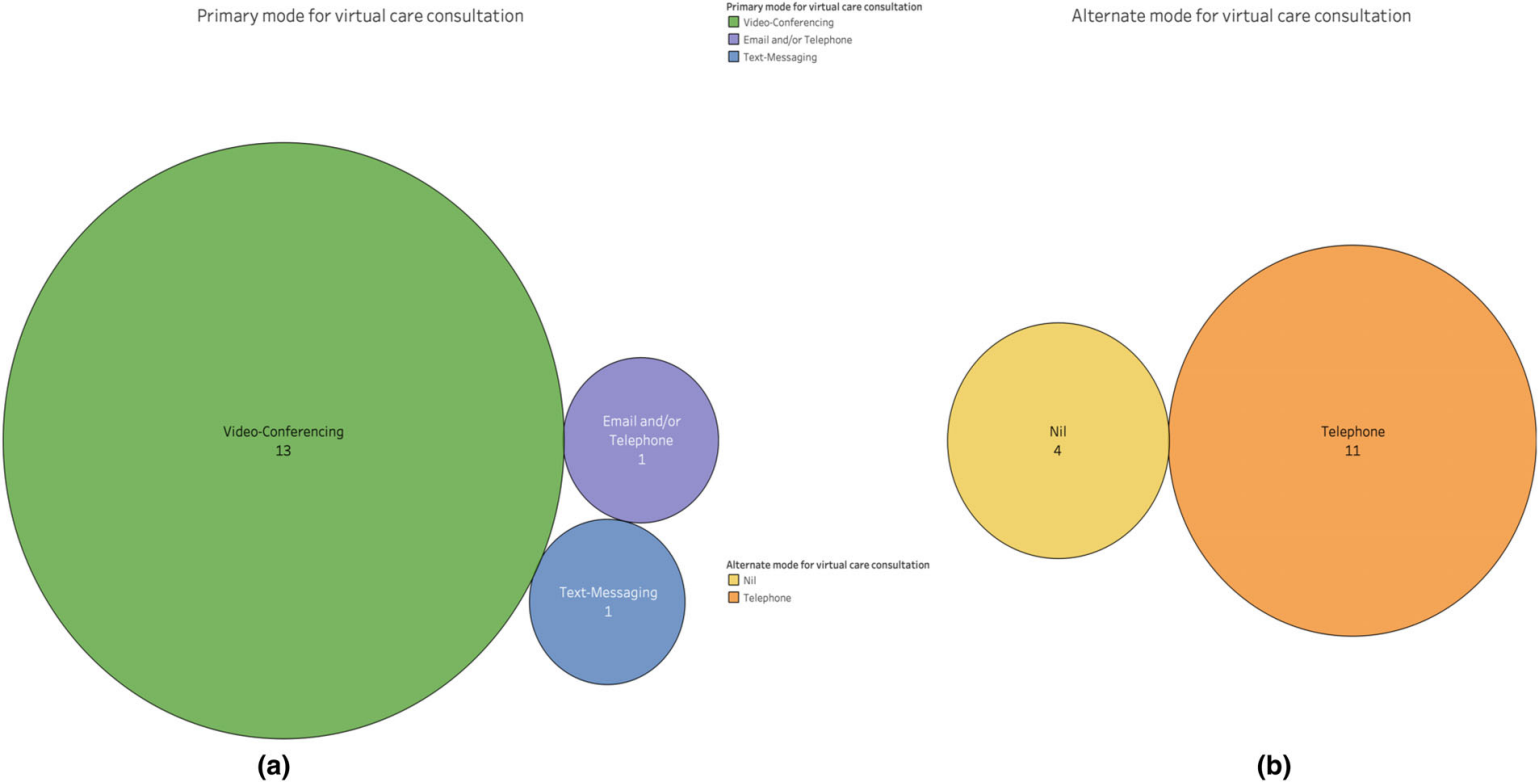
(a) Primary mode of system for virtual care consultation of included studies and (b) alternate mode of system for virtual care consultation of included studies.

**FIGURE 5:**
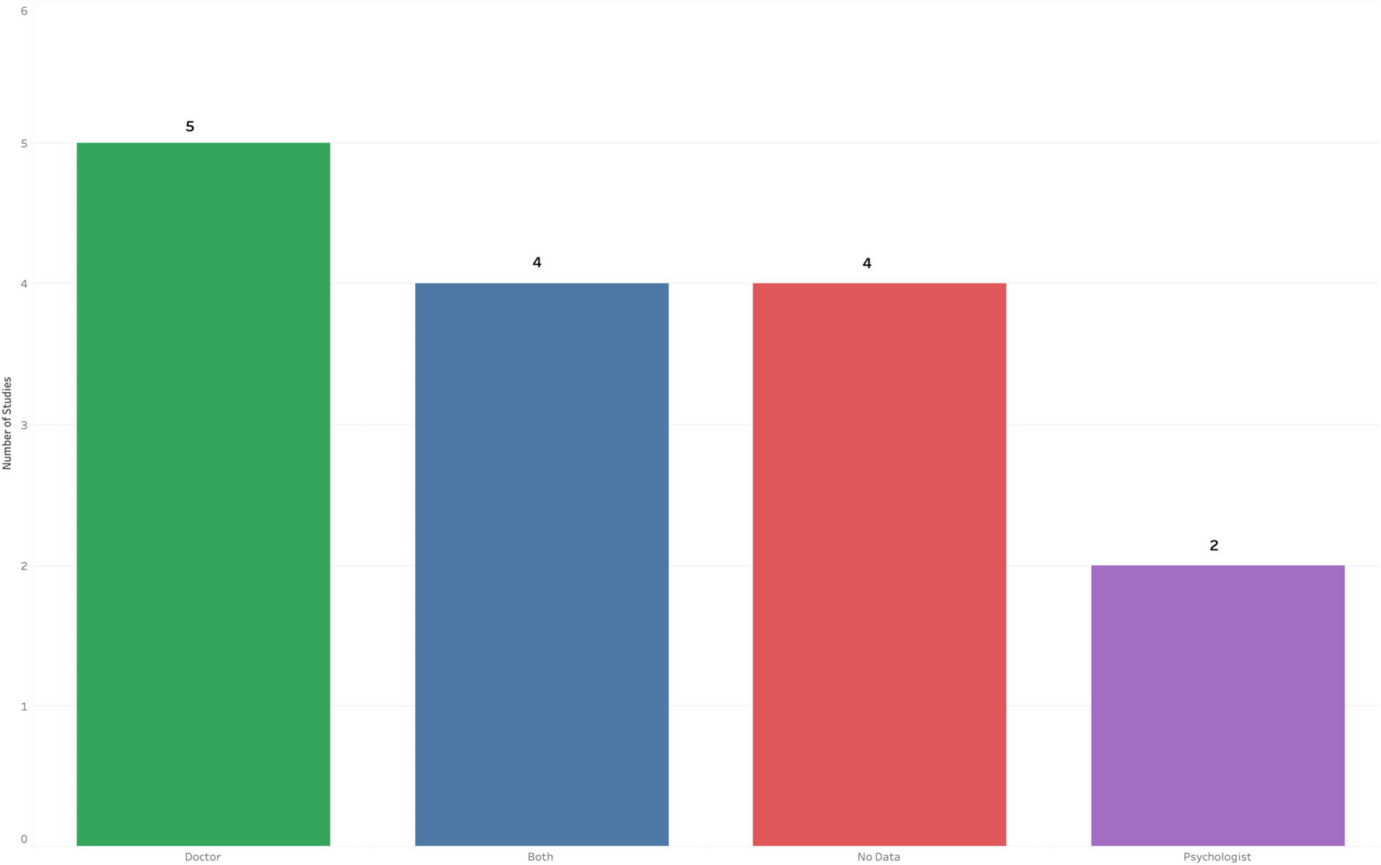
Designation of healthcare professional leading the consultation in the included studies.

The primary mode of virtual consultation was performed via videoconferencing (13 studies) with 11 studies demonstrating a fall‐back option of telephone consultations (Figure 4). Of the specific videoconferencing tools utilized, only one study utilized the widely‐used Zoom platform, with the majority of studies utilizing in‐house VC platforms (Table 5). The predominant HCWs for the VCs were doctors (5/15), while both nurses and doctors were involved in 4/15 studies, with psychologists leading in 2/15 and no data for 4 studies. Only 3/15 studies reported concurrent sexually transmitted infection (STI) screening; however, 12/15 studies did not display this data (Figure 5).

#### PLHIV and VC attendance

Twelve (12/15) studies reported on the total number of participants with completed and cancelled/DNA visits (Table 6). For these 12 studies, there were 5799 participants in VC, of which 4914 (84.74%) completed their VCs, with only 687 (11.85%) either cancelling or not attending their VCs.

#### Acceptability, satisfaction and adherence of PLHIV with HIV‐VC

Five (5/15) studies reported outcomes on user acceptability of VC, with a mean of 79.54% (interquartile range [IQR] 16.45% and standard deviation [SD] 9.98%) reporting VC as acceptable (i.e., in circumstances where patients did not have VC available; however, binarily stated [“Yes or No”] as to whether they would be happy for future VC). Five (5/15) studies reported satisfaction and adherence rates with 85% (IQR 10.5% and SD 14.5%) satisfied with VC and 86.90% fully adherent to ART (IQR 21.95% and SD 20.60%). Eight (8/15) studies reported the proportion of users with an undetectable HIV viral load (<50 copies/mL) with 87% of VC participants virally suppressed (Table 6).

### Narratives on models‐of‐care

These were studies reported as descriptive narratives or interventions without qualitative or quantitative results. Eight (8/20) models of care were described as part of a trial; the remainder were commentaries on real‐world implementation of virtual care. The COVID‐19 pandemic prompted all the described services to develop virtual care for the first time and included VC for hard‐to‐reach groups, such as individuals in prison (*n* = 1) or those living in rural areas (*n* = 3), or to continue care during the COVID‐19 pandemic (*n* = 8).

A variety of virtual care modalities were described, including phone (*n* = 4), video (*n* = 13) and two‐way messaging by SMS (*n* = 1), instant message or email (*n* = 1), with one unclear modality. All the telehealth interventions initiated as part of RCTs and implementation studies were opt‐in with informed consent, as would be expected.

In the analysis of descriptive implementation reviews, only two studies [35] explicitly mentioned providing patients with a choice, with one also formally obtaining consent for virtual care. Another study [40] noted that patients consented upon logging into the online portal, but it remained unclear whether patients were offered a choice before this point. The majority of observational and commentary publications did not clearly indicate whether patients were given the option to choose virtual care. Notably, three studies [25] reported a shift of all routine patient care to virtual platforms, suggesting a lack of patient choice; however, it is important to note that these changes were primarily described in the context of adaptations during the COVID‐19 pandemic. Of the clinics which did not offer patients a choice of in‐person or virtual care, six also described no provision for those who may experience digital exclusion due to lack of mobile phone or internet connection [39]. In other studies, this was accounted for either in exclusion criteria [41] or in steps made to counteract possible digital exclusion. For example, other services gave phones and went to great lengths to adapt their service to the needs of their more vulnerable patients, who did not engage with virtual care, contacting them by various means and conducting home visits. One study [28] mentioned providing satellite sites with an internet connection to address service‐based inequity.

Six studies detailed instances of virtual care wherein patients were situated in a purposefully arranged and controlled environment, such as a local health clinic or a prison clinic room. In these settings, the presence of an in‐person nurse was a common practice to facilitate consultations and conduct examinations. Three of these clinics also employed digital examination equipment, such as Bluetooth stethoscopes, capable of transmitting heart sounds to a remote clinician for further analysis.

Among these virtual care modalities, six publications highlighted the platforms for interacting with patients. These platforms were purpose‐built for healthcare applications and adhered to national healthcare data and communication regulations. Interestingly, Zoom was utilized as an alternative platform in only one study [22]. The platforms adopted varied in functionality, with some integrating messaging and video capabilities alongside features tailored for HIV patient education and results viewing. This diversity in platform features showcases the adaptability and customization potential of virtual care solutions in catering to the unique needs of HIV patient populations.

Six studies mentioned staff training, but the quality of this varied, with one using written guidance only [25] and others providing training sessions [35, 38] or training sessions in tandem with dedicated IT [20] or physician‐champion support [28]. Only one study [41] mentioned doing an initial pilot on the VC. Only four publications mentioned patient training, and this also varied hugely. One study [25] verbally explained the privacy risks of remote consultation only, one [35] provided written information and one [33] provided in‐person training as part of an initial visit.

### Qualitative data results

Five qualitative research papers describing attitudes to HIV‐VC were identified and are described in Table 4. All the studies were conducted in the USA except for one study conducted in Europe [32]. Three studies [30, 36, 40] focused on patient participants only, one [42] focused on health providers (specifically management personnel) and one [32] focused on both patient (client) and health provider (physician) participants.

The three major themes that have emerged from these studies are detailed in the following three sections.

#### Barriers to telehealth (remote health services)

All the studies except Junkins et al. (2021) [30] have delved into participants' perspectives about barriers to adopting or implementing VC. Among the concerns raised, security emerged as a prominent issue for patients, who feared potential exposure of their personal information due to hacking or technological failures. Privacy concerns were also expressed, particularly in finding suitable environments for remote interactions. Both HCWs and patient participants expressed that digital literacy posed a significant barrier, especially for those unfamiliar with the platforms, highlighting this as a major concern for first‐time users. Participants articulated that virtual interactions have limitations, particularly in the absence of physical examinations, leading to potential oversights in assessing patients' well‐being, such as detecting weight loss. Resource availability, encompassing access to smartphones, computers and network connectivity, was identified as another barrier. Additionally, health providers pinpointed bureaucratic issues within organizational contexts, citing delays in care delivery. In some instances, challenges arose in obtaining client information and signatures for essential forms due to the lack of in‐person contact, underscoring bureaucratic barriers to effective telehealth implementation.

In one study [41], it was that the infrastructure for telecare was already established before the pandemic. However, its utilization was hindered by reimbursement issues. This situation underwent a transformation when, at the onset of the pandemic, the Office of Medical Assistance Programs issued guidance permitting telehealth reimbursement for Medicaid patients and private insurers promptly adopted similar policies. Several papers emphasize the significance of swift COVID‐19‐related policy adjustments in the USA as crucial for instigating virtual care.

#### Facilitators to telehealth

All studies except Yelverton et al. (2021) [42] emphasized factors facilitating usage of telehealth. Participants highlighted the significance of convenience, emphasizing the cost savings from travel and the time saved, especially for those residing at a distance from health facilities. This convenience was particularly beneficial for individuals who were feeling well and stable, without complications necessitating in‐person clinic visits. Additionally, some participants noted that telehealth reduced stigma and anxieties associated with physically meeting physicians or encountering community members and acquaintances at clinics, potentially leading to inadvertent disclosure of their status. Some patients reported diminished anxiety related to face‐to‐face meetings with physicians fostered a sense of comfort among participants, encouraging them to disclose more information. For some patients, being physically distant from the physician eliminated the intimidation factor associated with being in the same room. However, physicians expressed concerns about telehealth diminishing empathy as they found that they gleaned more about a patient's background during face‐to‐face interactions, a nuance often lost in digital platforms. On the positive side, participants appreciated the flexibility of choosing the environment or platform to conduct telehealth sessions, which provided a sense of security and increased the likelihood of utilizing these services.

#### Suggested strategies for promoting virtual healthcare

Two studies [30, 32] did not propose any strategies for promoting telehealth. Nevertheless, both patient participants and physicians emphasized the importance of thorough orientation on how these platforms operate. Such orientation was deemed crucial in instilling confidence and proficiency in navigating through the diverse telehealth platforms. Patient participants specifically recommended the provision of essential resources such as smartphones and emphasized the need for enhanced security measures on the platforms.

### Risk of bias review of quantitative studies and quality of qualitative studies

In quantitative studies, eight had low risk of bias with a high NOS score of ≥7, four had medium risk of bias with NOS score of 5–6, and six had high risk of bias with scores <4 (Table 7). One study was not analysed for risk of bias as it was an expert commentary on standards for HIV‐VC without an appropriate study design and hence would not be appropriate for its study design. The two RCTs had a very low risk of bias, with NOS scores of 9, due to their study design. However, it is to be noted that the NOS score is not specifically designed for RCTs but rather for observational studies generally. The NOS score was utilized to standardize the risk of bias scores across quantitative studies.

**TABLE 7:** Risk of bias (Newcastle–Ottawa score) of studies – quantitative and implementation studies.

Reference	Type of study	Adequate case definition	Representativeness of cases	Selection of controls	Definition of controls	Representative of exposed cohort	Selection of non‐exposed cohort	Ascertainment of exposure	Demonstration that outcome of interest was not present at start of study	Comparability on the basis of the design or analysis	Ascertainment of exposure	Same method of ascertainment for participants	Nonresponse rate	Assessment of outcome	Was follow‐up long enough for outcomes to occur	Adequacy of follow‐up of cohorts	Total score
Auchus et al. (2021)	Cohort	NA	NA	NA	NA	⋆			⋆	⋆	NA	NA	NA	⋆		⋆	5
Badowski et al. (2012)	Cohort	NA	NA	NA	NA	*					NA	NA	NA	⋆			2
Brody et al. (2021)	Descriptive Implementation Review/Model of care	NA	NA	NA	NA	⋆		⋆			NA	NA	NA	⋆			3
Coppock et al. (2021)	Descriptive Implementation Review/Model of care	NA	NA	NA	NA	*					NA	NA	NA	⋆			2
Dandachi et al. (2020)	Cohort	NA	NA	NA	NA	⋆		⋆	⋆	⋆	NA	NA	NA	⋆	⋆	⋆	7
Dandachi et al. (2020)	Descriptive Implementation Review/Model of care	NA	NA	NA	NA	*					NA	NA	NA	⋆			2
Drummond et al. (2017)	Descriptive Implementation Review/Model of care	NA	NA	NA	NA			⋆						⋆			2
El‐Nahal et al. (2022)	Cohort	NA	NA	NA	NA	*		*	*	**				*	*	*	8
Guaraldi et al. (2021)	Expert Viewpoint	NA	NA	NA	NA	NA	NA	NA	NA	NA	NA	NA	NA	NA	NA	NA	NA
Hoberg et al. (2018)	Cohort	NA	NA	NA	NA	*		*	*	**				*	*	*	8
Jain et al. (2019)	Descriptive Implementation Review/Model of care	NA	NA	NA	NA	*		*	*	*				*	*		6
John et al. (2016)	Cohort	NA	NA	NA	NA	*	*	*	*	**	NA	NA	NA	*	*		8
Junkins et al. (2021)	RCT	*	*	*	*	NA	NA	NA	NA	**	*	*	*	NA	NA	NA	9
León et al. (2011)	RCT	*	*	*	*	NA	NA	NA	NA	**	*	*	*	NA	NA	NA	9
Rogers et al. (2020)	Descriptive Implementation Review/Model of care	NA	NA	NA	NA	*					NA	NA	NA	*			2
Saifu et al. (2012)	Cohort	NA	NA	NA	NA	*		*	*	*	NA	NA	NA	*	*		6
Salgado et al. (2021)	Cross‐sectional	NA	NA	NA	NA	*	*	*	*	*	NA	NA	NA	*	NA	NA	5
Trepka et al. (2022)	Cross‐sectional	NA	NA	NA	NA	*	*	*	*	**	NA	NA	NA	*	*	*	9
Wood et al. (2020)	Cross‐sectional	NA	NA	NA	NA	*	*	*	*	*				*		*	7

**Key**	Case–control or RCT only criteria for study risk of bias
	Cohort or cross‐sectional study criteria for study risk of bias

Abbreviations: NA, not available; RCT, randomized controlled trail.

All qualitative studies were systematically reviewed using the five criteria defined by Stenfors' framework [16] (Table 4). All five qualitative studies distinctly articulate their research questions, methods and data analysis procedures. However, one [36] lacks the presentation of its theoretical basis for analysis. The clarity in methodological delineation enables potential replication of these studies elsewhere, and the incorporation of direct quotes in findings reinforces the credibility of the collected data. Nevertheless, the generalizability of the studies is constrained, primarily due to the limited sample size employed, with one study [32] being an exception. Additionally, reflexivity is addressed to a minimal extent, with only three studies acknowledging that data collection was conducted by experienced researchers.

## DISCUSSION

From this mixed‐methods systematic review we can note that there is heterogenicity of VC for PLHIV. From our review we can determine that PLHIV do generally accept, value and are satisfied with VC. This is similar to studies in people without HIV for other chronic medical conditions, such as in people with diabetes [43], chronic kidney disease [44], cancer [45, 46, 47, 48], mental health conditions [49, 50, 51], bone and joint infections [52] and for genetic and psychological counselling [53] with positive patient outcomes and acceptability in both the Global North [54] and South [55] countries in patients, caregivers and healthcare staff [56, 57, 58, 59].

VC seems particularly useful in people who have achieved HIV viral suppression and have good adherence to treatment. With the majority of PLHIV in VC maintaining viral suppression, VC in PLHIV could have broader implications of viral load for therapeutic efficacy to improve quality of life. Various virtual care modalities were described, including phone, video and two‐way messaging, by SMS, instant message or email. It is to be noted that the majority of studies utilized in‐house VC platforms rather than readily available VC software such as Zoom, MS Teams or Skype. This may be due to information governance and security concerns which prompt researchers and HIV teams to develop in‐house platforms that have validated secured networks. In addition, we noted not all VC models discussed testing for STIs, which is recommended by national HIV guidelines [5]. This contrasts with other VC systems such as Australia's country‐wide initiative and expansion of outpatient parenteral antimicrobial therapy (OPAT) in combination with VC [52, 60, 61]. This combined model of VC care led to improved, safe, cost‐effective, clinical outcomes particularly for patients residing in remote and geographically isolated locations [52, 60, 61], albeit it was rolled across a single country in a high‐income setting. These diverse HIV‐VC healthcare systems seen in this review may reduce transferability of VC into various countries and a standardized VC model may improve patient care and outcomes globally. However, a universally applicable ‘gold standard’ for virtual care models does face numerous challenges, as its development relies on the unique circumstances of each healthcare setting. To advance this, HIV‐VC guidance may benefit under the leadership and assistance of the World Health Organization, similar to its guidance for setting goals for eliminating HIV and hepatitis C [62, 63, 64].

From the key qualitative studies, we found that three overarching themes emerge. First, there were barriers to telehealth encompassed by security concerns with challenges in creating private virtual spaces, digital literacy issues, restricted physical examination capabilities, and resource availability challenges. Second, facilitators of telehealth include the convenience of reduced travel costs and time, especially for distant patients, as well as diminished stigma and anxiety associated with face‐to‐face interactions. However, physicians express concerns about reduced empathy in virtual consultations. Lastly, strategies for promoting telehealth highlight the importance of orientation to platform operation for both clients and physicians, together with the provision of essential resources like smartphones and enhanced platform security.

This review underscores the existence of significant data gaps, highlighting the need for a more thorough investigation into the virtual provision of HIV care. The quantitative analysis conducted in this study faced limitations due to a relatively restricted sample size, as a noteworthy proportion of the included studies failed to comprehensively collect measurable outcome data. Future research endeavours should specifically target these gaps to advance our understanding and improve the quality of evidence. A particular emphasis in future research should be placed on areas such as HIV monitoring, switching ART and long‐term complications. Addressing these data gaps will enhance our understanding of the nuances in virtual care for PLHIV and contribute to the development of more comprehensive and patient‐centric virtual healthcare strategies. Further studies and data can lead to a clearer understanding of the optimal mix of face‐to‐face and VC that could be applied in the clinic setting with interim guidance being utilized to ensure confidentiality and patient safety [65].

As technology continues to play an increasingly integral role in healthcare delivery, bridging these gaps becomes imperative for ensuring the optimal delivery of care to individuals with HIV and improving their overall health outcomes.

As most studies predominantly represent the Global North, there is also a concern that findings may lack full representativeness with only one study based primarily in the Global South (Nigeria) [29]. Further studies need to be performed in Global South sites and, in doing so, researchers can enhance the evidence base for VC effectiveness, ensuring that findings are robust and applicable to a broader patient population. There is also a crucial need for increased research focus on rural populations in the Global South. Further investigations should specifically target underrepresented and marginalized populations, encompassing studies in incarcerated settings and among individuals who inject drugs. Expanding the scope of virtual care research to include these diverse populations will contribute to a more comprehensive understanding of virtual care's effectiveness and challenges in addressing these communities' unique needs. Our review included non‐English studies in the search strategy, however we excluded these studies only post abstract screening (three studies – one in Russian and two in Spanish) due to lack funding of translation services [66]. The inclusion of these non‐English studies may in the future provide a broader perspective and potentially different approaches to HIV‐VC. However, researchers would have to ensure that the translation and interpretation of such studies could be adequately performed to ensure the results would be comparable [67].

From the literature we can observe virtual care's evolution in response to the COVID‐19 pandemic, delineating a distinction between trial‐based interventions and those implemented in real‐world scenarios. Notably, the pandemic acted as a catalyst for initiating virtual care services, either as a novel endeavour or to sustain healthcare provision during the crisis. In light of the COVID‐19 pandemic's impact on the rapid adoption of virtual care, the majority of VC interventions in the current review were implemented in response to the pandemic, making it essential to evaluate the sustained effectiveness and integration of VC into routine healthcare beyond the crisis. This reassessment should explore the long‐term impact of VC on patient outcomes, healthcare accessibility and provider–patient relationships. Additionally, post‐COVID reassessment should address the scalability and generalizability of virtual care models.

Targeting specific demographics, such as individuals in prison or those residing in rural areas, virtual care embraced diverse modalities including phone, video and various messaging platforms. The approach to patient consent varied, with opt‐in models for interventions stemming from RCTs and implementation studies, while some descriptive reviews lacked clarity on patient choice. Concerns arose regarding patient choice, particularly during the pandemic, where some studies indicated a unilateral shift to virtual care. Additionally, several clinics failed to address issues of digital exclusion, potentially exacerbating disparities in access. Mitigation strategies were implemented in certain studies to counteract digital exclusion, ranging from providing devices and adapting services for vulnerable patients to conducting home visits. Some studies even established satellite sites with internet access to address service‐based inequities.

Six studies detailed virtual care settings in health clinics or prison clinic rooms, often facilitated by in‐person nurses and featuring digital examination equipment transmitting data to remote clinicians. Platform diversity emerged as a notable aspect, with the majority adhering to national regulations, and only one study deviating by employing Zoom. The variable landscape of staff and patient training was evident, with approaches ranging from written guidance to dedicated IT support. Patient training, mentioned in a limited number of studies, utilized methods such as verbal explanations, written information and in‐person sessions during initial visits. Notably, only one study referenced the importance of conducting an initial pilot for virtual care, emphasizing the need for strategic testing and refinement in the implementation process. This underscores the multifaceted nature of virtual care adoption, encompassing issues of consent, patient choice, digital inclusion, platform diversity and the crucial role of training initiatives for both staff and patients.

The risk of bias assessment demonstrated heterogeneity in the methodological quality of all included studies. Notably, only eight studies were deemed to have a low risk of bias, reflecting robust research designs with NOS scores exceeding 7. This variability underscores the importance of critically evaluating the methodological rigour of virtual care studies in the context of HIV, emphasizing the need for standardized approaches and heightened scrutiny in future research endeavours. Moreover, it prompts a consideration of the potential impact of bias on study outcomes and reinforces the call for greater methodological consistency in the exploration of virtual care for diverse populations, especially in underrepresented regions like the Global South.

## CONCLUSIONS

This systematic review provides evidence that VC for PLHIV is acceptable, feasible and effective in achieving good clinical outcomes. The majority of the studies reported on the delivery of general HIV virtual care, with videoconferencing as the primary mode of virtual consultation. There are multiple models of care and a ‘gold standard’ needs to be considered to ensure PLHIV are appropriately reviewed in VC settings. Further research is needed to investigate the impact of VC on vulnerable populations and to identify strategies to increase patient engagement in virtual care.

## AUTHOR CONTRIBUTIONS

Conceptualization: HZF, LW, JPT, JA, CM. Data curation: HZF, LW, CM, JPT. Formal analysis: HZF, LW, CM, SJIMS. Funding acquisition: JPT, JA. Investigation: HZF, LW, CM, JPT. Methodology: HZF, LW, CM, JPT. Supervision: JPT, JA. Validation: HZF, LW, CM, JPT, SJIMS. Visualization: HZF. Roles/Writing – original draft: HZF, LW, CM, JPT. Writing – review and editing: all authors.

## FUNDING INFORMATION

This work was supported by Gilead UK and Ireland Fellowship and the British HIV Association (BHIVA). HZF was supported with funding from the Wellcome Trust (grant reference: 227516/Z/23/Z). The SHARE collaborative was supported with funding from Bart's Charity.

## CONFLICT OF INTEREST STATEMENT

HZF has received travel grants and honoraria from Janssen‐Cilag; travel grants from Gilead Sciences, ViiV Healthcare Ltd, European Society of Clinical Virology, British HIV Association and the European AIDS Clinical Society. JPT has received speaker fees from Gilead Sciences, and conference registration fees from ViiV healthcare. In October 2023, JPT became a paid employee of ViiV healthcare.

## ETHICS STATEMENT

Not required as a literature review.

## CONSENT FOR PUBLICATION

All authors gave their consent for publication.

## Supporting information


**Data S1.** Supporting Information.

## Data Availability

All the data for this study will be made available upon reasonable request to the corresponding author.
