# Supplementary material for: Virtual care pathways for people living with HIV: A mixed‐methods systematic review
Source: HIV Med. 2024 Sep 17;26(1):44–69. doi: 10.1111/hiv.13701 (PMC11725418; doi:10.1111/hiv.13701)
Supplement: Supplementary file 1 — Data S1. Supporting Information. [file HIV-26-44-s001.docx]

**HIV Care From a Distance: Designing and Delivering Effective Person-Centred Care in the Context Of SARS-Cov-2**

| Database | Provider | No. Results |
| --- | --- | --- |
| Medline | OVID | 1114* |
| Embase | OVID | 1359* |
| Emcare | OVID | 649* |
| CINAHL | EBSCO | 614 |
| Cochrane CENTRAL | Wiley | 407 |

*after duplicates removed within OVID: 1806

All searches completed by Pip Divall, Clinical Librarian on 4^th^ May 2022

[Pip.divall@uhl-tr.nhs.uk](mailto:Pip.divall@uhl-tr.nhs.uk)

Please reference this search as:
*Divall, P. Evidence Search: HIV Care from a distance. Leicester: University Hospitals of Leicester NHS Trust Libraries and information services, 04/05/2022.*

**Embase <1974 to 2022 May 03>**

**Ovid Emcare <1995 to 2022 Week 17>**

**Ovid MEDLINE(R) ALL <1946 to May 03, 2022>**

1 hiv/ or hiv-1/ or hiv-2/ 367778

2 ("acquired immunodeficiency syndrome" or "acquired immunedeficiency syndrome" or "acquired immuno‐deficiency syndrome" or "acquired immune‐deficiency syndrome").mp. 118398

3 "HIV/AIDS".mp. 97321

4 HIV.mp. 939420

5 PLWHA.mp. 3226

6 or/1-5 1026331

7 Remote Consultation/ 23478

8 (teleconsult* or tele-consult*).mp. [mp=ti, ab, hw, tn, ot, dm, mf, dv, kf, fx, dq, nm, ox, px, rx, ui, sy] 21652

9 exp Telemedicine/ 121598

10 Videoconferencing/ 12061

11 (telehealth* or tele-health*).mp. [mp=ti, ab, hw, tn, ot, dm, mf, dv, kf, fx, dq, nm, ox, px, rx, ui, sy] 42196

12 (telemedicine* or tele-medicine*).mp. [mp=ti, ab, hw, tn, ot, dm, mf, dv, kf, fx, dq, nm, ox, px, rx, ui, sy] 104577

13 (tele-monitor* or telemonitor*).mp. [mp=ti, ab, hw, tn, ot, dm, mf, dv, kf, fx, dq, nm, ox, px, rx, ui, sy] 10283

14 (telemanagement or tele-management).mp. [mp=ti, ab, hw, tn, ot, dm, mf, dv, kf, fx, dq, nm, ox, px, rx, ui, sy] 265

15 (telecare* or tele-care*).mp. [mp=ti, ab, hw, tn, ot, dm, mf, dv, kf, fx, dq, nm, ox, px, rx, ui, sy] 3075

16 "remote care".mp. 1017

17 (mobile adj health*).mp. 23391

18 "computer mediated therapy".mp. 10

19 video conferenc*.mp. [mp=ti, ab, hw, tn, ot, dm, mf, dv, kf, fx, dq, nm, ox, px, rx, ui, sy] 4358

20 (virtual adj2 (appointment* or consult* or clinic*)).mp. [mp=ti, ab, hw, tn, ot, dm, mf, dv, kf, fx, dq, nm, ox, px, rx, ui, sy] 4735

21 (video adj2 (appointment* or consult* or clinic*)).mp. 5419

22 or/7-21 192944

23 6 and 22 3037

24 exp Human immunodeficiency virus/ 377450

25 ("acquired immunodeficiency syndrome" or "acquired immunedeficiency syndrome" or "acquired immuno‐deficiency syndrome" or "acquired immune‐deficiency syndrome").mp. 118398

26 "HIV/AIDS".mp. 97321

27 exp acquired immune deficiency syndrome/ 262116

28 HIV.mp. 939420

29 PLWHA.mp. 3226

30 or/24-29 1104726

31 exp teleconsultation/ 24277

32 Remote consultation.mp. [mp=ti, ab, hw, tn, ot, dm, mf, dv, kf, fx, dq, nm, ox, px, rx, ui, sy] 7103

33 (teleconsult* or tele-consult*).mp. [mp=ti, ab, hw, tn, ot, dm, mf, dv, kf, fx, dq, nm, ox, px, rx, ui, sy] 21652

34 exp telemedicine/ 121598

35 videoconferencing/ 12061

36 (telehealth* or tele-health*).mp. [mp=ti, ab, hw, tn, ot, dm, mf, dv, kf, fx, dq, nm, ox, px, rx, ui, sy] 42196

37 (telemedicine* or tele-medicine*).mp. [mp=ti, ab, hw, tn, ot, dm, mf, dv, kf, fx, dq, nm, ox, px, rx, ui, sy] 104577

38 (tele-monitor* or telemonitor*).mp. [mp=ti, ab, hw, tn, ot, dm, mf, dv, kf, fx, dq, nm, ox, px, rx, ui, sy] 10283

39 (telemanagement or tele-management).mp. [mp=ti, ab, hw, tn, ot, dm, mf, dv, kf, fx, dq, nm, ox, px, rx, ui, sy] 265

40 (telecare* or tele-care*).mp. [mp=ti, ab, hw, tn, ot, dm, mf, dv, kf, fx, dq, nm, ox, px, rx, ui, sy] 3075

41 "remote care".mp. 1017

42 (mobile adj health*).mp. [mp=ti, ab, hw, tn, ot, dm, mf, dv, kf, fx, dq, nm, ox, px, rx, ui, sy] 23391

43 "computer mediated therapy".mp. 10

44 video conferenc*.mp. 4358

45 (virtual adj2 (appointment* or consult* or clinic*)).mp. [mp=ti, ab, hw, tn, ot, dm, mf, dv, kf, fx, dq, nm, ox, px, rx, ui, sy] 4735

46 (video adj2 (appointment* or consult* or clinic*)).mp. [mp=ti, ab, hw, tn, ot, dm, mf, dv, kf, fx, dq, nm, ox, px, rx, ui, sy] 5419

47 or/31-46 193175

48 30 and 47 3122

49 23 use medall 1114

50 48 use oemezd 1359

51 48 use emcr 649

52 49 or 50 or 51 3122

53 remove duplicates from 52 1806

| # | Query | Results |
| --- | --- | --- |
| S1 | (MH "Human Immunodeficiency Virus+") | 10,975 |
| S2 | ("acquired immunodeficiency syndrome" or "acquired immunedeficiency syndrome" or "acquired immuno‐deficiency syndrome" or "acquired immune‐deficiency syndrome") | 19,519 |
| S3 | ""HIV/AIDS"" | 26,174 |
| S4 | HIV | 125,312 |
| S5 | PLWHA | 604 |
| S6 | S1 OR S2 OR S3 OR S4 OR S5 | 133,476 |
| S7 | (MH "Remote Consultation") | 2,819 |
| S8 | "teleconsult* or tele-consult*" | 1 |
| S9 | (MH "Telemedicine") | 14,909 |
| S10 | (MH "Videoconferencing") | 2,556 |
| S11 | (MH "Telehealth") OR "telehealth* or tele-health*" | 11,973 |
| S12 | telemedicine* or tele-medicine* | 24,583 |
| S13 | tele-monitor* or telemonitor* | 1,022 |
| S14 | telemanagement or tele-management | 0 |
| S15 | telecare* or tele-care* | 687 |
| S16 | "remote care" | 143 |
| S17 | mobile N1 health* | 5,076 |
| S18 | "computer mediated therapy" | 0 |
| S19 | video conferenc* | 682 |
| S20 | (virtual n2 (appointment* or consult* or clinic*)) | 950 |
| S21 | video n2 (appointment* or consult* or clinic*) | 1,239 |
| S22 | S7 OR S8 OR S9 OR S10 OR S11 OR S12 OR S13 OR S14 OR S15 OR S16 OR S17 OR S18 OR S19 OR S20 OR S21 | 37,998 |
| S23 | S6 AND S22 | 614 |

CENTRAL

ID Search

#1 MeSH descriptor: [HIV] explode all trees

#2 "acquired immunodeficiency syndrome" or "acquired immunedeficiency syndrome" or "acquired immuno‐deficiency syndrome" or "acquired immune‐deficiency syndrome"

#3 "HIV/AIDS"

#4 HIV

#5 PLWHA

#6 {or #1-#5}

#7 MeSH descriptor: [Remote Consultation] explode all trees

#8 teleconsult* or tele-consult*

#9 MeSH descriptor: [Telemedicine] explode all trees

#10 MeSH descriptor: [Videoconferencing] explode all trees

#11 telehealth* or tele-health*

#12 telemedicine* or tele-medicine*

#13 tele-monitor* or telemonitor*

#14 telemanagement or tele-management

#15 telecare* or tele-care*

#16 "remote care"

#17 mobile near/1 health*

#18 "computer mediated therapy"

#19 video conferenc*

#20 virtual near/2 (appointment* or consult* or clinic*)

#21 video near/2 (appointment* or consult* or clinic*)

#22 {or #7-#21}

#23 #7 and #22
